# Air Pollution, Body Composition, and Vascular Age in Southern Switzerland: A Cross-Sectional Population Study

**DOI:** 10.3390/jcm14196971

**Published:** 2025-10-01

**Authors:** Matteo Pasini, Martina Zandonà, Maria Luisa Garo, Claudio Bozzini, Francesca Cinieri, Rosaria Del Giorno, Luca Gabutti

**Affiliations:** 1 Istituto Cardiocentro, Ente Ospedaliero Cantonale, 6900 Lugano, Switzerland; matteo.pasini@eoc.ch; 2 Family Medicine Service, Ente Ospedaliero Cantonale, 6900 Lugano, Switzerland; francesca.cinieri@eoc.ch; 3 Biostatistics Unit, Mathsly Research, 00128 Rome, Italy; marilu.garo@mathsly.it; 4 Swiss Federal Institute for Forest, Snow and Landscape Research WSL, 8903 Birmensdorf, Switzerland; claudio.bozzini@wsl.ch; 5 Angiology Service, Ente Ospedaliero Cantonale, 6500 Bellinzona, Switzerland; rosaria.delgiorno@eoc.ch; 6 Università della Svizzera Italiana, 6900 Lugano, Switzerland

**Keywords:** air pollution, vascular age, pulse wave velocity, body composition, population, cross-sectional, Switzerland

## Abstract

**Background:** Air pollution is a well-established risk factor for cardiovascular and metabolic diseases. Although Southern Switzerland is considered a relatively low-pollution area, levels of nitrogen dioxide (NO_2_) and particulate matter (PM_10_) still exceed the latest WHO air quality guidelines. This study aimed to assess the association between long-term exposure to air pollutants, vascular aging, and body composition in a Swiss population sample. **Methods:** A cross-sectional analysis was conducted on 1202 participants from the Ticino Epidemiological Stiffness Study (2017–2018). Vascular health was assessed via pulse wave velocity (PWV), used to estimate vascular age. Body composition was evaluated through bioimpedance analysis, yielding fat mass index (FMI), body cellular mass (BCM), and body cellular mass index (BCMI). Individual exposure to NO_2_ and PM_10_ was estimated, using geocoded residential data and environmental monitoring records from 2002 to 2017. Statistical models were adjusted for major cardiovascular risk factors. **Results:** Higher exposure to NO_2_ was significantly associated with increased vascular age (mean delta age: +0.53 years in the high exposure group) and adverse body composition markers, including higher FMI and lower BCM/BCMI. These associations remained significant after adjusting for confounders. PM_10_ showed weaker associations, significant only in unadjusted models. **Conclusions:** Even in a relatively clean environment, exposure to NO_2_ is linked to early vascular aging and unfavorable body composition. These findings reinforce the need for stricter air quality standards and underline the importance of continuous environmental health surveillance, even in regions considered low risk.

## 1. Introduction

Atmospheric pollution is a global health threat caused by both natural phenomena and human activities. The magnitude of the effect on health has been estimated to be around 300,000 premature deaths for the year 2019 in Europe [1], on a total population of around 446 million people. Despite a significant improvement in European air quality since 1990, in 2021, 97% of the people living in urban areas were still exposed to concentrations of fine particulate matter above the thresholds of the 2021 World Health Organization guidelines [2].

The understanding of the impact of air pollution on health has constantly increased from the industrial revolution in XIX and XX centuries, progressively bringing the scientific knowledge to public attention. From the evidence of massive excess death rates during the “Great Smog” in 1952 in London [2], researchers have accumulated a wide body of evidence leading to progressively stricter recommendations on acceptable air pollution levels [3]. The last-released WHO guidelines were based on several studies showing a negative effect on health of air pollution even at concentrations lower than previously recommended [4,5,6,7,8,9].

A wide range of human activities contribute to air pollution: agriculture; energy supply; the manufacturing and extractive industry; road and non-road transport; waste (via decomposition and burning); and residential, commercial, and institutional consumption [1]. Air pollutants can be divided into solid phase pollutants (PM_10,_ PM_2.5_, PM_0.1_) and gaseous pollutants (NO_x_, NO_2_, O_3_, SO_2_). These substances are associated with a wide variety of negative effects on the nervous, cardiovascular, respiratory, reproductive, and digestive systems [3,10].

In particular, the association between air pollution and overt cardiovascular diseases (ischemic heart disease [11], CV mortality and stroke [12,13,14], hypertension [15], heart failure [16], and peripheral artery disease [17]) as well as subclinical indicators of vascular aging [18,19], BMI, and body composition [20,21] has been investigated widely. Exposure to both air pollutants in the solid phase and gases is associated with worsening of the aforementioned outcomes, even with some differences in the strength of evidence and exposure duration trigger. The strongest evidence is between higher levels of pollution in the short- and long-term and all-cause CVD mortality and morbidity, stroke, hypertension, and ischemic heart disease (IHD) [12]. In a longitudinal study of 28 million people based in seven European countries, one of which was Switzerland, the association between long-term low-level air pollution and non-accidental mortality was investigated: even at concentrations lower than recommended by 2005 WHO guidelines, an increase of 5 µg/m^3^ of PM_2.5_ and 10 µg/m^3^ NO_2_ was associated with a HR of 1.078 (1.046–1.111) and 1.049 (1.024–1.075), respectively [4]. Regarding body mass (BMI), a positive association was found with PM_10_, PM_2.5_, and O_3_ exposure [18,19], while fat mass index (FMI) was associated only with PM_10_ [19]. Concerning preclinical atherosclerosis, many studies demonstrated an association between pulse wave velocity (PWV) or reflected waves (AI, AP), as well as the thickness of the intima and media in the carotid artery and the exposure to particulate matter or gaseous pollutants [17,20,21].

In Southern Switzerland, in recent decades, a significant reduction in emissions has been achieved: in particular, a fall from 30% (for O_3_ levels) to 60% (for SO_2_ levels) has been observed since 1990, when monitoring began systematically [22]. The progress in technology with cleaner combustion techniques is mostly responsible for this reduction, whilst a significant reduction in consumption itself has not been achieved. Nevertheless, the mean level of the main polluting agents remains higher than the limit values proposed by the 2021 WHO guidelines in the most anthropized area of our region [3,22]. Moreover, it must be noted that the cited guidelines provide stricter cutoffs that are still not adopted by Swiss regulations, which refers to the previous edition of 2005 [3,23].

The pollution levels in Southern Switzerland are influenced by a complex orography between the Italian Padan plain and the Alps, with anthropized areas located in valleys and along lakeshores. Social and economic factors, namely the international highway transport-axis-relying central Europe and Italy and trans-border work with subsequent use of private and public mean of transport, contribute to local emissions related to road and non-road transport. Industry, agriculture and residential, commercial, and institutional consumption are also among the major local determinants. The proximity of the Padan plain with its peculiar atmospheric characteristics, namely the thermal inversion phenomenon, and high emissions from human activities is also an important factor [24]. Those facts strongly influence the concentration of pollutants, providing the possibility to study its effects in low and relatively high concentrations in a small geographical area.

**Figure 1:**
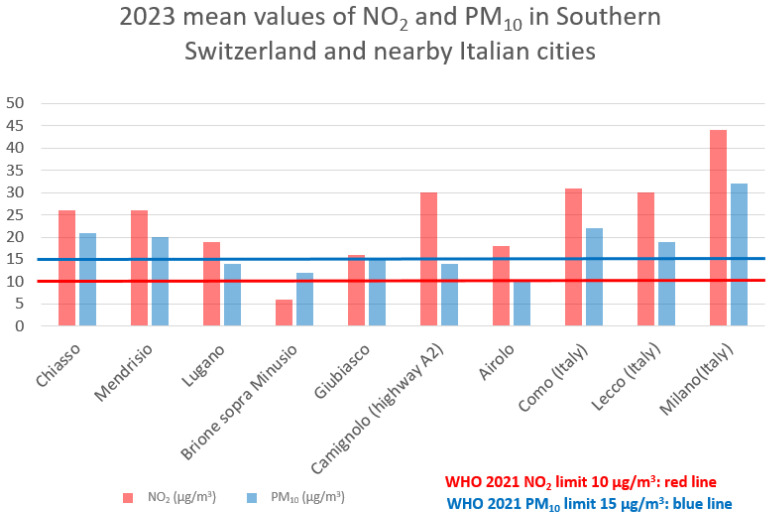
Mean values of NO_2_ and PM_10_ in Southern Switzerland and nearby Italian cities.

In comparison with proximity regions, the mean yearly values of the most polluted sampling station of Southern Switzerland (Chiasso) superposes with values of the nearby Italian region of Como but are significantly lower than the extremely urbanized area of Milan [22,25]. Conversely, the less polluted areas (Brione sopra Minusio for NO_2_, Airolo for PM_10_, PM_5_, and NO_3_) show concentrations that meet the WHO 2021 Guideline recommendations (Figure 1).

The aim of our research is to establish if air pollution in Southern Switzerland, despite levels that mostly conform to Swiss regulation, at the population level, is associated with significant alterations in clinical and subclinical cardiovascular disease indicators and body composition.

## 2. Materials and Methods

This study relies on a cross-sectional evaluation derived from a population-based research project, the Ticino Epidemiological Stiffness Study (TEST study), conducted between 2017 and 2018 in the Italian-speaking region of Switzerland (Canton Ticino). The TEST study was carried out in accordance with the principles of the 1964 Helsinki Declaration and its later amendments. Approval was granted by the Swiss ethics committee (CE 3115-2016-01718), and written informed consent was obtained from all participants.

In the original cohort, 1202 individuals were enrolled. Each participant underwent a blood sample collection to determine serum glucose, HbA1c, creatinine, low-density lipoprotein cholesterol, high-density lipoprotein cholesterol, triglycerides, total cholesterol, and cystatin-C levels. In addition, all participants were fitted with a Mobil-O-Graph (I.E.M. GmbH, Stolberg, Germany) ambulatory blood pressure monitor (ABPM), to record 24 h blood pressure values. Measurements were performed every 30 min during daytime and once per hour overnight. Monitoring was scheduled to be on working days, and participants were instructed to maintain their usual daily activities. Arterial stiffness was evaluated through pulse wave velocity (PWV) using a SphygmoCor tonometric device (AtCor Medical Pty Ltd., Sydney, Australia).

Body composition was further assessed by a BIA 101 bioimpedance analyser (Akern Srl, Pisa, Italy), which provided estimates of fat mass (FM) and body cellular mass (BCM)—the metabolically active fraction of the body. From these measures, the fat mass index (FMI) and body cellular mass index (BCMI) were calculated by normalizing for height squared.

Participants were considered at risk if they presented at least one of the following conditions: current smoking, diabetes, history of cardiovascular disease, chronic kidney disease (CKD) stage ≥3 according to KDIGO classification, LDL cholesterol of ≥4.4 mmol/L, hypertension, or ongoing treatment with lipid-lowering and/or antihypertensive agents, as well as metabolic syndrome. Metabolic syndrome was defined according to the NCEP ATP III criteria, that is, the presence of three or more of the following: a waist circumference of >101.6 cm in men or >88.9 cm in women, blood pressure of >130/85 mmHg, fasting triglycerides of >1.69 mmol/L, fasting high-density lipoprotein (HDL) cholesterol of <1.03 mmol/L in men or <1.29 mmol/L in women, and fasting glucose of >5.5 mmol/L. When fasting glucose was not available, a HbA1c value of ≥5.7% was considered as the threshold. Estimated glomerular filtration rate (eGFR) was calculated using the 2021 CKD-EPI creatinine–cystatin equation. Laboratory tests were performed using a Cobas analyser (Roche Diagnostics, Basel, Switzerland).

**Figure 2:**
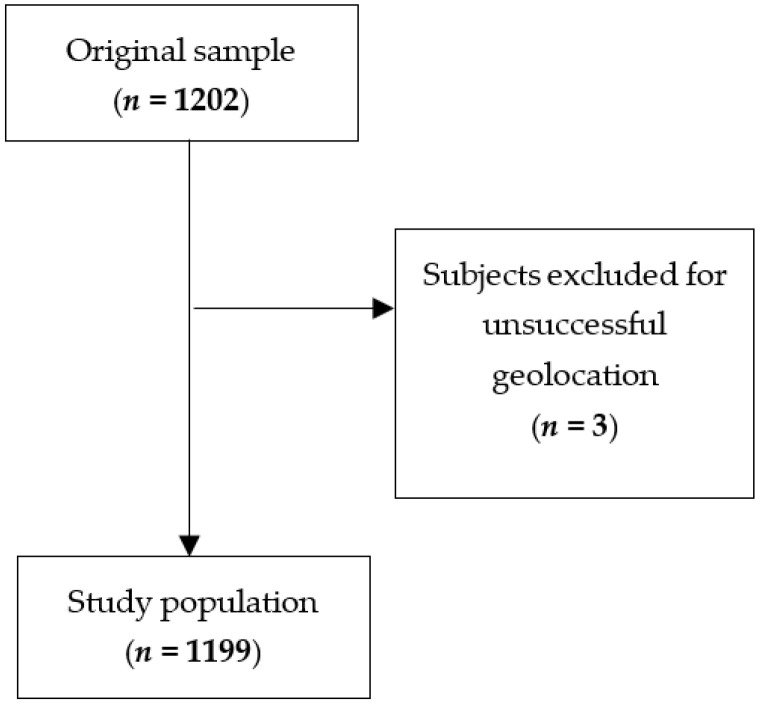
Flowchart showing the participants’ selection procedure.

To analyze the correlation between the health status of the population sample and the levels of air pollutants (NO_2_ and PM_10_) in their area of residence, geospatial and patient address data were integrated. Annual average concentrations of NO_2_ and PM_10_ from 2002 to 2017 were obtained from raster maps of the Canton of Ticino. Patient addresses were geocoded using the GIS software ArcGIS Pro 3.1.0 to obtain World Geodetic System 1984 coordinates, which were then converted to the Swiss CH1903/LV03 reference system to match the raster data. Of the 1202 addresses, 1199 were successfully geolocated (Figure 2). Five incomplete or missing addresses were manually approximated using cadastral maps, while two addresses with no usable information were excluded. NO_2_ and PM_10_ values for each location and year were extracted using the translator library GDAL 3.7.2 in batch mode provided by the GIS software QGIS 3.28.11. The resulting dataset (25,242 records) was processed using R version 4.3.1 to generate both a complete dataset with annual values and a summary dataset with per-subject statistics. This method enabled the integration of environmental data with individual-level records based solely on address information.

Since the data from the TEST study were collected between 2017 and 2018, we decided to use the mean pollution values from 2002 to 2017. Subsequently, the population was divided into three exposure categories based on pollutant levels (low, moderate, and high).

### Statistical Analysis

Descriptive statistics are expressed as frequency and percentage for categorical and dichotomous variables, while mean and standard deviation are used for quantitative variables. To estimate vascular age, we used a linear regression model, calculated using the data of subjects without cardiovascular risk factors participating in the study, in which chronological age was the dependent variable and pulse wave velocity was the independent variable. With this approach, vascular age was estimated indirectly by interpreting it as the predicted chronological age corresponding to a given PWV value.

Univariate linear regression models were used to assess the impact of pollution on vascular age, delta age (i.e., the difference between vascular age and chronological age), and fat mass index (FMI). Further regression analyses were conducted on other body composition parameters. All models were subsequently adjusted for a covariate that classified each patient according to the presence or absence of at least three risk factors. We chose to include this single covariate in the classification of patients according to their risk factors, rather than assessing each risk factor individually, because we needed a robust and less susceptible variable to random variation that could capture the overall risk. The assumptions of each linear regression model were verified.

Differences in vascular age, delta age, and FMI in subjects with and without risk factors and in subjects exposed to different levels of NO_2_ were also analyzed using the U Mann–Whitney test and the Kruskal–Wallis with Dunn procedure, respectively. Statistical significance was set at 5% (*p* < 0.05). All statistical analyses were performed using STATA 18 (StataCorp., College Station, TX, USA).

## 3. Results

Patients’ characteristics are summarized in Table 1.

**Figure 3:**
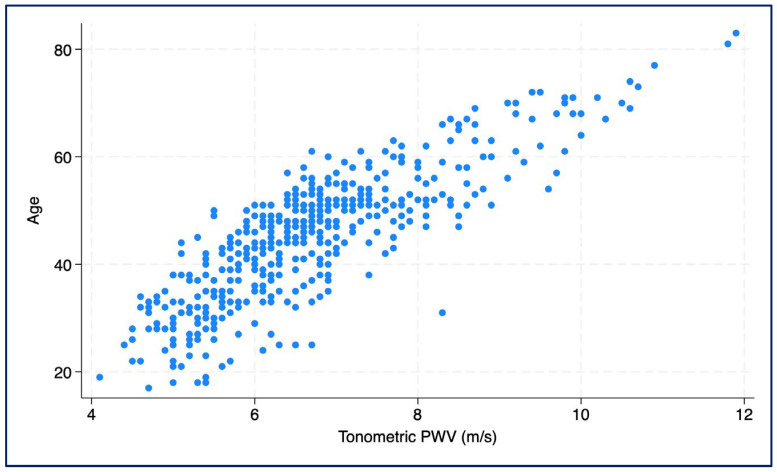
Scatter plot showing the association between chronological age and tonometrically measured pulse wave velocity (PWV).

A vascular age model was developed based on the tonometric PWV, taking into account subjects without major cardiovascular risk factors. This showed that each 1 m/s increase in PWV corresponded to an average increase in chronological age of 7.6 years (β = 7.58; 95% CI: 7.13–8.03; *p* < 0.001; R^2^ = 0.694) (Figure 3).

**Figure 4:**
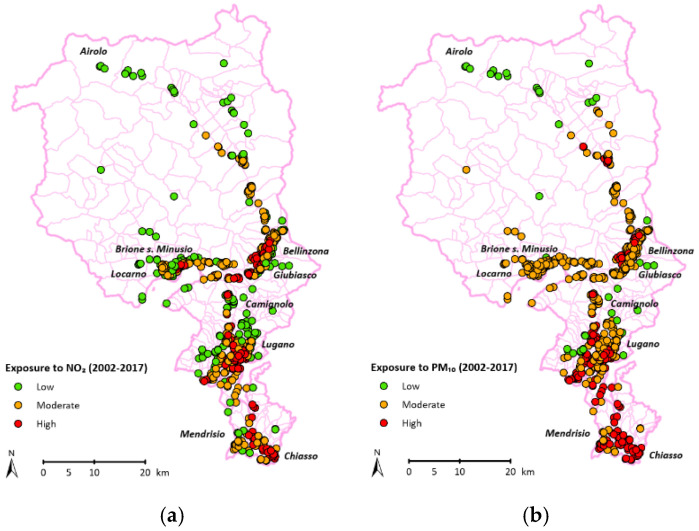
Exposure of participants to NO_2_ (**a**) and PM_10_ (**b**).

The mean pollution values from 2002 to 2017 were NO_2_: 22.88 ± 4.44 µg/m^3^ [min: 7.69, max: 42.75] and PM_10_: 23.96 ± 3.07 µg/m^3^ [min: 10.75, max: 36.25]). We divided the population into three exposure categories based on pollutant levels (Figure 4), specifically as follows:

NO_2_:

- Low 16.77 ± 2.97 µg/m^3^ (range: 7.69–19.93)
- Moderate: 22.57 ± 1.45 µg/m^3^ (range: 20.13–25.0)
- High: 27.58 ± 2.53 µg/m^3^ (range: 25.06–42.75)

PM_10_:

- Low: 15.35 ± 2.65 µg/m^3^ (range: 10.75–19.75)
- Moderate: 23.19 ± 1.11 µg/m^3^ (range: 20.0–24.94)
- High: 26.96 ± 2.31 µg/m^3^ (range: 25.0–36.25)

**Figure 5:**
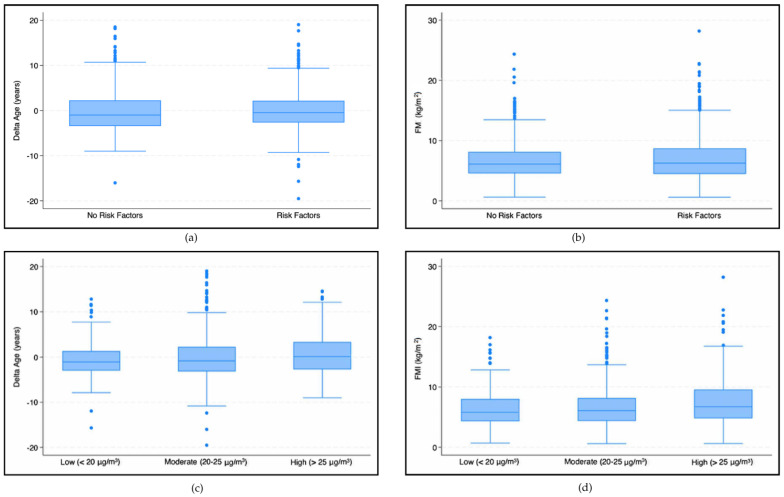
Box plots—The first two graphs (**a,b**) show differences in the delta age (**a**) and FMI (**b**) in patients with and without risk factors. The subsequent graphs show differences in the delta age (**c**) and FMI (**d**) in patients exposed to different NO_2_ concentration levels.

In our analysis we found significant associations between the exposure to the investigated pollutants and vascular age and body composition parameters. Higher FMI values were observed in patients exposed to higher levels of NO_2_ concentrations (7.40 ± 3.85 kg/m^2^) compared with those exposed to low (6.49 ± 3.09 kg/m^2^) and moderate (6.81 ± 3.59 kg/m^2^) NO_2_ concentrations (*p* < 0.001) (Figure 5d). The influence of NO_2_ concentration on FMI was confirmed in both unadjusted (β = 0.05, SE: 0.02, *p* = 0.053) and adjusted regressions (β = 0.05, SE: 0.02, *p* = 0.044) (Table 2).

NO_2_ concentrations also showed a negative effect on BCM (β = −0.16, Se: 0.05, *p* = 0.002) and BCMI (β = −0.04, SE: 0.01, *p* = 0.002). These reductions were also confirmed in the adjusted analysis, which showed that NO_2_ concentration had a negative effect on BCM (β = −0.16, SE: 0.05, *p* = 0.002) and BCMI (β = −0.04, SE: 0.01, *p* = 0.002).

Analyzing the effect of NO_2_ concentrations using our vascular age model, we found that high concentrations (>25 μg/m^3^) had a statistically significant higher mean delta age (0.53 ± 4.34 years) than those exposed to moderate (−0.05 ± 4.72 years) and low (−0.63 ± 3.90 years) NO_2_ concentrations (*p* < 0.001) (Figure 5c). An increase in NO_2_ concentration results in an increase in delta age (βNO_2_ = 0.08, SE: 0.03, *p* = 0.008). The adjusted analysis confirmed the trend (βNO_2_ = 0.08, SE: 0.03, *p* = 0.007).

Concerning solid phase pollutants, we found an effect of PM_10_ concentration on vascular age in the unadjusted analysis that was not confirmed in the adjusted analysis (Table 3).

## 4. Discussion

We conducted a secondary analysis on a vascular health population-based study (TEST study) carried out in Southern Switzerland to assess the effect of pollution in the area of residence.

We obtained pollution information from a local authorities’ database, detailing in particular the exposure to NO_2_ and PM_10_ from 2002 to 2017. Considering that the study only enrolled the adult population, we used the mean values of the entire observation period to estimate individual exposure.

Detailed characterization of the included subjects’ cardiovascular risk factors, 24 h ambulatory blood pressure monitoring, body composition, medications, and pulse wave velocity (PWV) were used to create adjusted models aimed at assessing the correlation with vascular health status. To assess the gap between expected and measured vascular age, we created a model of vascular aging based on the PWV in subjects without cardiovascular risk factors.

Significant associations were found using NO_2_ values. Keeping in mind that the 2021 WHO guidelines recommend a level below 10 μg/m^3^ but current local regulations adopt the 25 μg/m^3^ threshold of the previous 2005 document, we divided subjects according to their mean annual exposition to three different NO_2_ levels: high (>25.06 μg/m^3^), moderate (20.13–25.0 μg/m^3^), and low (7.69–19.93 μg/m^3^). The group exposed to the lower concentrations had significantly less fat mass and more body cellular mass, in both the adjusted and unadjusted analysis, suggesting a negative effect of pollution on body metabolism.

Concerning our model of vascular age, we found a negative effect of NO_2_ concentration on the estimated age based on pulse wave velocity, with subjects exposed to higher pollution levels being on average “1 year older” than the less exposed ones (0.53 ± 4.34 years vs. −0.63 ± 3.90 years).

Concerning PM_10_, we also noted a significant effect on vascular age but limited to the unadjusted model.

Interestingly, we observed a continuous negative correlation between the measured health parameters and pollution levels in the whole spectrum of the registered pollutant’s concentration, confirming, on a local basis, the appropriateness of the last WHO guidelines, recommending to lower the previous thresholds.

As for the limitations, our study is a cross-sectional analysis and subsequently cannot provide information about outcomes. To address this gap in knowledge, we expect to conduct a longitudinal study concerning the same topic.

Another limit of our study concerns the evaluation of the individual exposure. We chose to use pollution levels in the residential area of the subjects, defined by their address and not their workplace. Furthermore, our values, according to majority of international studies, only depict the outdoor situation. As to this point, some studies have suggested that there can be a significant difference between outdoor and indoor levels of pollution, with the latter experiencing higher concentrations [26,27,28], but unfortunately, no wide-scale data on indoor pollution is currently available in our region. Moreover, additional pollutants and factors that were not evaluated in the present study, such as PM_0.01_ and PM_2.5_, and individual genetic susceptibilities cannot be excluded as potential contributors to the variations we observed.

Last but not least, a part of the studied population passes a relevant percentage of their time working at an unspecified distance from their place of residence and possibly in a higher anthropized area, as most of the industrial plants and offices are located in the polluted areas of the region. An underestimation of the exposure is subsequently probable, suggesting that the magnitude of the difference in health parameters observed between high and low exposure groups could be even more clinically significant.

## 5. Conclusions

The unfavorable effect of air pollution, and NO_2_ levels in particular, on vascular health and body composition can be recognized, in a significant sample of the population, even in a low-polluted area such as Southern Switzerland. In particular, a significant difference can be seen between the inhabitants of areas complying with the new, stricter limits proposed by the WHO and those in force in Switzerland. Those living in an area with a higher NO_2_ concentration have on average an older vascular age and have more adipose and less lean tissues than the rest of the population. Data from this cross-sectional survey have already been highlighted in previous epidemiological studies; the magnitude of the consequences and the long-term implications, however, have to be evaluated in a longitudinal study.

The importance of ensuring low levels of air pollution for the health of the population is emphasized once again.

## Figures and Tables

**Table 1:** Patients’ characteristics.

Variable	Mean or Frequency	% or SD
**No. of patients**	1202	
**Sex, *n* (%)**		
Female	672	(56.0)
Male	528	(44.0)
**Age (age), mean (SD)**	50.4	(13.7)
**Country of birth, *n* (%)**		
Suisse	816	(68.0)
Other country	384	(32.0)
**Years of residence in Ticino, mean (SD)**	41.4	(19.0)
**Diseases and lifestyle, *n* (%)**		
Previous CVD	39	(3.3)
Hypertension	192	(16.1)
Hypercholesterolemia	173	(14.5)
Diabetes	26	(2.2)
Smoking	236	(19.6)
CKD	24	(2.0)
Metabolic syndrome	104	(8.7)
**Medications, *n* (%)**		
Antihypertensives	188	(15.8)
Hypolipidemic	176	(14.8)
**Body Composition, mean (SD)**		
FM (kg)	19.6	(10.0)
BCM (kg)	29.2	(7.8)
BCMI (kg/m^2^)	10.1	(2.1)
FMI (kg/m^2^)	6.9	(3.6)
BMI (kg/m^2^)	25.1	(4.4)
**Blood pressure values, mean (SD)**		
Systolic pressure 24 H (mm/Hg)	119.1	(11.9)
Diastolic pressure 24 H(mm/Hg)	74.2	(8.8)
Daily Systolic pressure (mm/Hg)	121.9	(12.3)
Daily Diastolic pressure (mm/Hg)	76.9	(9.1)
Dipping (%)	8.3	(6.9)
**PWV Tonometric (m/s), mean (SD)**	7.4	(1.7)
Vascular age *		
Mean (SD) (years)	50.5	(13.0)
Patients with vascular age ≤ chronological age, *n* (%)	640	(58.0)
Patients with vascular age > chronological age, *n* (%)	463	(42.0)
Patients with at least three risk factors **, *n* (%)	716	(59.6)

* Vascular age was determined on a sample of 1103 patients because of some missing values in the PWV values. ** Patients were classified as at risk when three or more of the following factors were present: smoking, diabetes, previous cardiovascular disease, CKD of ≥3, LDL of ≥4.4 mmol/L, hypertension, the use of hypolipidemic drugs, the use of hypertensive drugs, and metabolic syndrome. FFM: fat-free mass. BCM: body cellular mass. BCMI: body cellular mass index. FMI: fat mass index.

**Table 2:** Unadjusted and adjusted regression for body composition parameters.

	Unadjusted Analysis			Adjusted for Risk Factors *					
	BCM	BCMI	FMI	BCM	BCMI	FMI	BCM	BCMI	FMI
**NO_2_ Concentration**	−0.16 † (0.05)	−0.04 † (0.01)	0.05 (0.02)	−0.16 † (0.05)	−0.04 † (0.01)	0.05 † (0.02)			
**PM_10_ Concentration**	−0.10 (0.08)	−0.02 (0.02)	0.02 (0.04)				−0.10 (0.08)	−0.02 (0.02)	0.02 (0.04)
**Risk factors** (Ref. no risk factors) *				−0.06 (0.47)	−0.08 (0.13)	0.26 (0.22)	−0.02 (0.47)	−0.06 (0.13)	0.24 (0.22)

BCM: body cellular mass. BCMI: body cellular mass index. FMI: fat mass index. * Patients were classified as at risk when three or more of the following factors were present: smoking, diabetes, previous cardiovascular disease, CKD of ≥3, LDL of ≥4.4 mmol/L, hypertension, the use of hypolipidemic drugs, the use of hypertensive drugs, and metabolic syndrome. The variable risk factor is a dichotomous variable that takes a value of 1 if the patient has at least three of the risk factors listed above and 0 otherwise. The results are given as coefficients β and (standard error). †: *p* < 0.05.

**Table 3:** Unadjusted and adjusted regression models for delta age.

	Unadjusted Analysis	Adjusted for Risk Factors	
	Delta Age *	Delta Age *	
NO_2_ Concentration (μg/m^3^)	0.08 † (0.03)	0.08 † (0.03)	
PM_10_ Concentration (μg/m^3^)	0.06 (0.04)		0.07 (0.04)
Risk factors (Ref. no risk factors) **		0.22 (0.27)	0.18 (0.27)

* Delta age = vascular age—chronological age. ** Patients were classified as at risk when three or more of the following factors were present: smoking, diabetes, previous cardiovascular disease, CKD of ≥3, LDL of ≥4.4 mmol/L, hypertension, the use of hypolipidemic drugs, the use of hypertensive drugs, and metabolic syndrome. The variable risk factor is a dichotomous variable that takes a value of 1 if the patient has at least three of the risk factors listed above and 0 otherwise. The results are given as coefficients β and (standard error). †: *p* < 0.05.

## Data Availability

Study data are obtainable from the authors upon reasonable request.

## Abbreviations

The following abbreviations are used in this manuscript:

BP	Blood pressure
CVD	Cardiovascular disease
ABPM	Ambulatory blood pressure monitoring
PWV	Pulse wave velocity
PM	Particulate matter
FM	Fat mass
FMI	Fat mass index
BCM	Body cellular mass
BCMI	Body cellular mass index
BMI	Body mass index
